# Limitations and recommendations for bibliometric insights into breast cancer organoid chips: trends & emerging areas- a commentary

**DOI:** 10.1097/JS9.0000000000003098

**Published:** 2025-07-23

**Authors:** Weibo Sun, Haoran Wang, Chun Lou

**Affiliations:** a Department of Breast Surgery, Harbin Medical University Cancer Hospital, Harbin City, Heilongjiang Province, China; b Graduate School of Harbin Medical University, Harbin City, Heilongjiang Province, China

*Dear Editor,*

The paper “Bibliometric Insights into Breast Cancer Organoid Chips: Trends & Emerging Areas” by Tian *et al*^[1]^ provides a systematic bibliometric analysis of research trends in breast cancer organoid technology, mapping key developments using tools like CiteSpace and VOSviewer. While the study offers a comprehensive overview of publication trends, collaborations, and emerging hotspots—such as the shift from basic organoid culture optimization to clinical applications like drug screening and personalized therapy—it has several limitations that hinder its potential impact. Below, we outline these shortcomings and propose actionable improvements to strengthen future research in this field. The current paper is compliant with the TITAN Guidelines 2025—governing declaration and use of AI^[2]^.

First, narrow data source and potential bias: The study relies exclusively on the Web of Science (WoS) database, ignoring other major databases like Scopus, PubMed, or regional repositories such as CNKI for Chinese literature. This risks overlooking significant contributions, particularly from non-English publications, and may skew the analysis toward Western or high-impact journals^[3]^. For instance, China’s dominance in publication output (74 articles) might be even more pronounced if local databases were included.

Second, superficial keyword analysis: While the keyword co-occurrence network identifies terms like “breast cancer,” “microfluidics,” and “tumor microenvironment,” the paper fails to delve into their contextual significance. For example: how do keywords like “triple-negative breast cancer” correlate with specific technological advancements such as vascularized chips? Are there gaps between frequently studied mechanisms and clinical applications? A deeper thematic analysis, such as natural language processing (NLP) of abstracts, could reveal subtler trends.

Third, lack of interdisciplinary metrics: The study mentions the integration of organoids with CRISPR, AI, and microfluidics but does not quantify these intersections bibliometrically. Co-citation analysis across disciplines such as bioengineering or patent literature could highlight translational pathways and innovation bottlenecks^[4]^.

Forth, overlooking negative or null findings: The paper notes challenges in vascularization but does not cite studies that attempted—and failed—to replicate functional vasculature in organoid chips. Addressing such gaps would provide a more balanced view of the field’s challenges.

Fifth, weak clinical and policy linkages: Despite highlighting clinical applications such as drug screening, the study does not connect bibliometric trends to real-world adoption. Metrics like the number of organoid-based clinical trials, FDA approvals, or industry partnerships would better demonstrate the technology’s translational progress.

Last, poor visualization clarity: Figures like the keyword cluster map are overly complex and lack annotations, making it difficult to extract actionable insights. Simplified visuals such as heatmaps of keyword evolution over time or interactive dashboards would improve accessibility. Therefore, to improve future bibliometric studies in this field, researchers should broaden data sources by incorporating multiple databases and grey literature such as preprints and patents to reduce selection bias and capture a more comprehensive global research landscape^[5]^; enhance thematic depth through NLP analysis of abstracts and full texts to identify sub-themes such as immune-organoid interactions and their correlations with citations or funding trends, potentially revealing understudied areas ripe for innovation; quantify interdisciplinary impact by mapping co-citations between journals in oncology, bioengineering, and computational biology to identify cross-disciplinary hubs; address reproducibility and ethics by dedicating sections to analyzing publications on organoid reproducibility challenges and ethical debate to better inform policymakers and funding agencies, and improve visual communication by replacing dense network diagrams with clearer visualizations such as timelines of keyword bursts paired with technological milestones and geospatial maps of collaboration networks to highlight regional strengths and weaknesses^[6]^.

In sum, while Tian et al.’s study provides a useful snapshot of breast cancer organoid research, its reliance on a single database, superficial keyword interpretation, and lack of translational metrics limit its utility. By expanding data sources, deepening thematic analysis, and linking trends to clinical or industrial outcomes, future bibliometric work could better guide researchers, funders, and policymakers in advancing this promising field.

## Data Availability

This is a commentary about a published article, an there is no original or analysed secondary data in the manuscript.

## Research registration unique identifying number (UIN)

This is a commentary about a published article, thus is not registered.

## Declarations

Artificial Intelligence (AI), deepseek R1 was used only for checking form grammatical errors and revising sentence structures. No patient data was provided to AI. All the authors supervised and reviewed AI output, and the authors take responsibility for the integrity of the content affected.
